# Supplementary material for: Geriatric Oncology in Portugal: Where We Are and What Comes Next—A Survey of Healthcare Professionals
Source: Geriatrics (Basel). 2022 Sep 6;7(5):91. doi: 10.3390/geriatrics7050091 (PMC9498886; doi:10.3390/geriatrics7050091)
Supplement: Supplementary file 1 [file geriatrics-07-00091-s001.zip › geriatrics-1882100-supplementary.pdf]

geriatrics

Supplementary Table S1: Survey questions and response options.

**1. Does the hospital where you work offer any geriatric oncology and/or geriatrics consultations?**<br>a. Yes, but only geriatric oncology consultations<br>b. Yes, but only geriatrics consultations<br>c. Yes, it offers both consultations<br>d. No<br>e. I don't know
**2. In the medical oncology service of the hospital where you work, is there a doctor specifically dedicated to geriatric oncology?**<br>a. Yes<br>b. No<br>c. I don't know
**3. Does the hospital where you work have specific management protocols for elderly cancer patients?**<br>a. Yes<br>b. No<br>c. I don't know
**4. From your clinical practice, do you perceive the number of elderly cancer patients (>70 years) has increased?**<br>a. Yes<br>b. No<br>c. I don't know
**5. In your opinion, do elderly cancer patients need more specific care when compared to younger patients?**<br>a. Yes<br>b. No<br>c. I have never thought about this subject
**6. Do you feel the need for assessment scales for elderly cancer patients, in addition to ECOG-Performance status and Karnofsky, to help you make treatment decisions?**<br>a. Yes

b. No

c. I have never thought about this subject

7. In your clinical practice, do you use any geriatric assessment/screening to evaluate the elderly cancer patients (even if they are not validated for Portuguese language)?

a. Yes. Please list the scales you regularly use

b. No

c. I have never heard about geriatric assessment

8. Do you think that more information and training in geriatric oncology is needed?

a. Yes

b. No

c. I have never thought about this subject

9. How do you think geriatric assessment could help you in your clinical practice? You can choose more than one option.

a. To detect frailty

b. To predict toxicity

c. To predict survival

d. To define a treatment strategy

e. To improve quality of life

f. I don't think geriatric assessment would help in my clinical practice

10. What do you think is important to develop in the field of geriatric oncology in Portugal? You can choose more than one option.

a. Creation of geriatric oncology Units

b. Systematic geriatric assessment in oncology services

c. Geriatricians to be part of multidisciplinary teams

d. Creation of study groups in geriatric oncology

e. Invest in training in geriatrics both at the undergraduate and postgraduate level

f. I don't believe anything is necessary

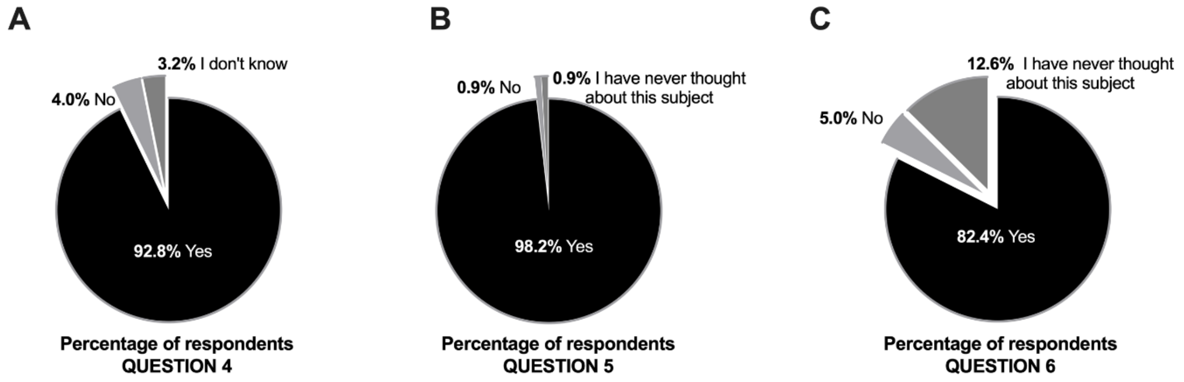

Supplementary Figure S1: Perceptions and challenging factors about the management of older patients with cancer. **(A)** From your clinical practice, do you perceive the number of elderly cancer patients (>70 years) has increased? **(B)** In your opinion, do elderly cancer patients need more specific care when compared to younger patients? **(C)** Do you feel the need for assessment scales for elderly cancer patients, in addition to ECOG-Performance status and Karnofsky, to help you make treatment decisions? (A,B,C) values are expressed as percentages, total number of answers (n=222).
